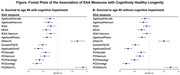# Do epigenetic age‐acceleration estimators predict survival to age 90 with preserved cognition? The Women's Health Initiative Memory Study (WHIMS)

**DOI:** 10.1002/alz70860_099854

**Published:** 2025-12-23

**Authors:** Andrea Z. LaCroix, Bowei Zhang, Linda K. McEvoy, Steve Nguyen, Ake Lu, Steve Horvath, Mark A. Espeland, Stephen R. Rapp, Adam Maihofer, Caroline Nievergelt, Susan M. Resnick, Kenneth Beckman, Aladdin H. Shadyab

**Affiliations:** ^1^ University of California San Diego, La Jolla, CA, USA; ^2^ Kaiser Permanente Washington Health Research Institute, Seattle, WA, USA; ^3^ Altos Labs, Cambridge, Cambridgeshire, United Kingdom; ^4^ Wake Forest University, Winston‐Salem, NC, USA; ^5^ National Institute on Aging, National Institutes of Health, Baltimore, MD, USA; ^6^ University of Minnesota Genomics Center, Minneapolis, MN, USA

## Abstract

**Background:**

Numerous epigenetic age‐acceleration estimators (EAA measures) have been shown to predict mortality, but none were trained specifically on longevity or preserved cognition. Importantly, little is known about whether these estimators can predict long survival with intact cognition.

**Methods:**

This analysis is based on 5293 women enrolled in the WHIMS born before February 28, 1934 (mean age=70.1 ±3.8 years) without prior mild cognitive impairment (MCI) or dementia at baseline (1995‐1998) who could have survived to age 90 by February 28, 2024 with data on their cognitive status within 2 years of their 90^th^ birthday. We examined associations of 14 epigenetic age‐acceleration estimators (Horvath age acceleration (AA), Hannum AA, AgeAccelPheno, AgeAccelGrim2, DunedinPACE, DNAmTelomereLength, plus 3 variations and 5 principle component (PC) versions of these clocks) in relation to survival to age 90 with preserved cognition (*n* = 1726, 33%) or without preserved cognition (adjudicated MCI or dementia, or self‐reported severe memory loss, *n* = 956, 18%) vs. the outcome of death before age 90 (*n* = 2611, 49%) using multinomial logistic regression. Models were adjusted for age, education, race, ethnicity, smoking, hormone trial arm, physical activity, body mass index, diabetes, CVD, cancer, and blood cell composition.

**Results:**

As shown in the Figure, associations in the predicted direction were observed for all of the accelerated age estimators in relation to survival to age 90 *with* cognitive impairment with the strongest associations observed for AgeAccelGrim2 (odds ratio (OR) = 0.67; 95% CI 0.60‐0.75) and PCGrimAge (OR=0.64; 95% CI 0.58‐0.70). Associations were in the same direction and of similar magnitude for survival to age 90 without cognitive impairment, except for the 2 IEAA variations which had 95% confidence intervals including 1. Associations appeared somewhat stronger for the PC versions of the estimators than for the original versions, for both longevity outcomes.

**Conclusions:**

EAA measures consistently predict survival to age 90 in older women, however they do not differentially predict survival with vs. without intact cognition. These findings support the further development of EAA measures trained on preserved cognitive status at advanced ages.